# Genome-Wide Investigation of DNA Methylation Marks Associated with FV Leiden Mutation

**DOI:** 10.1371/journal.pone.0108087

**Published:** 2014-09-29

**Authors:** Dylan Aïssi, Jessica Dennis, Martin Ladouceur, Vinh Truong, Nora Zwingerman, Ares Rocanin-Arjo, Marine Germain, Tara A. Paton, Pierre-Emmanuel Morange, France Gagnon, David-Alexandre Trégouët

**Affiliations:** 1 Sorbonne Universités, UPMC Univ Paris 06, UMR_S 1166, Team *Genomics & Pathophysiology of Cardiovascular Diseases*, Paris, France; 2 INSERM, UMR_S 1166, Team *Genomics & Pathophysiology of Cardiovascular Diseases*, Paris, France; 3 ICAN Institute for Cardiometabolism and Nutrition, Paris, France; 4 Division of Epidemiology, Dalla Lana School of Public Health, University of Toronto, Toronto, Canada; 5 Centre de Recherches du CHUM, Montréal, Canada; 6 The Centre for Applied Genomics and Program in Genetics and Genome Biology, The Hospital for Sick Children, Toronto, Ontario, Canada; 7 Aix-Marseille University, UMR_S 1062, Nutrition Obesity and Risk of Thrombosis, Marseille, France; 8 INSERM, UMR_S 1062, Nutrition Obesity and Risk of Thrombosis, Marseille, France; 9 Laboratory of Haematology, La Timone Hospital, Marseille, France; Medical University Hamburg, University Heart Center, Germany

## Abstract

In order to investigate whether DNA methylation marks could contribute to the incomplete penetrance of the FV Leiden mutation, a major genetic risk factor for venous thrombosis (VT), we measured genome-wide DNA methylation levels in peripheral blood samples of 98 VT patients carrying the mutation and 251 VT patients without the mutation using the dedicated Illumina HumanMethylation450 array. The genome-wide analysis of 388,120 CpG probes identified three sites mapping to the *SLC19A2* locus whose DNA methylation levels differed significantly (p<3 10^−8^) between carriers and non-carriers. The three sites replicated (p<2 10^−7^) in an independent sample of 214 individuals from five large families ascertained on VT and FV Leiden mutation among which 53 were carriers and 161 were non-carriers of the mutation. In both studies, these three CpG sites were also associated (2.33 10^−11^<p<3.02 10^−4^) with biomarkers of the Protein C pathway known to be influenced by the FV Leiden mutation. A comprehensive linkage disequilibrium (LD) analysis of the whole locus revealed that the original associations were due to LD between the FV Leiden mutation and a block of single nucleotide polymorphisms (SNP) located in *SLC19A2*. After adjusting for this block of SNPs, the FV Leiden mutation was no longer associated with any CpG site (p>0.05). In conclusion, our work clearly illustrates some promises and pitfalls of DNA methylation investigations on peripheral blood DNA in large epidemiological cohorts. DNA methylation levels at SLC19A2 are influenced by SNPs in LD with FV Leiden, but these DNA methylation marks do not explain the incomplete penetrance of the *FV* Leiden mutation.

## Introduction

Venous thrombosis (VT) is a common complex disease characterized by a sibling relative risk of ∼3 [1] and heritability estimates ranging from 30% to 60% [2], [3]. Contrary to other complex diseases, few new VT susceptibility genes were discovered by the recent waves of Genome-Wide Association Studies (GWAS) [4]. Established VT-associated genes collectively explain only about 5% of the disease heritability [2] and family history of VT remains an important risk factor despite adjustment for known variants [5], [6]. In addition, marked clinical variability is observed even in affected individuals from the same family and carrying the same mutation [6]. In particular, the penetrance of the *FV* Leiden mutation (i.e. *F5* R506Q or rs6025T/C), one of the major VT genetic risk factors present in about 5% of the general population, is quite low, only 10% of heterozygotes and 80% of homozygotes develop VT in their lifetime, with varying severity among affected individuals. These observations suggest that additional genetic and non-genetic factors contribute to the incomplete penetrance of FV Leiden and the clinical heterogeneity VT, as well as idiopathic VT.

Several lines of evidence support the role of DNA methylation marks as contributing factors in complex human diseases, including thrombosis-related disorders [7]–[11]. For example, quantitative risk factors for VT such as body-mass-index [12] and levels of von Willebrand factor [13], Factor VIII [14], and homocysteine [15] have been associated with DNA methylation marks. Further, lifestyle and environmental VT risk factors, such as smoking and air pollution, have been associated with methylation levels in genes relevant to VT pathophysiological mechanisms [16]–[18]. Until recently, such investigations were restricted to experimental models or small study samples, and restricted to candidate genomic regions.

The recent enthusiasm for agnostic investigations of methylation marks in peripheral blood DNA as a mean to investigate complex disease etiology and to generate novel mechanistic hypotheses is justified [19]–[21]. First,genome-wide methylation arrays, such as the Illumina HumanMethylation450 bead array, are now widely recognized as robust and efficient tools for epidemiological studies aiming at identifying methylation marks at CpG sites associated with environmental and genetic risk factors [12], [22], [23]. Second, biobanked peripheral blood DNA has been shown to be a robust and practical model for epidemiological epigenetic investigations [12], [24]–[26]. Third, evidence of peripheral blood DNA methylation marks as surrogates for methylation marks at other disease relevant tissues and cell types are increasingly emerging [12], [23], [24]. As whole blood DNA methylation levels reflect the average level resulting from the epigenetic state at different cell types, the identification of DNA methylation marks in peripheral blood cells may point out to novel biological mechanisms that subsequently can be validated in the principal effector cell types where stronger associations are expected [12]. Finally, and specific to this study, DNA from peripheral blood originates mainly from leukocytes, which are key effector cells for both coagulation and inflammation, the two principal pathophysiological mechanisms underlying VT.

In the current work, we hypothesized that DNA methylation marks contribute to the incomplete penetrance of the FV Leiden mutation. We undertook a DNA methylome-wide association scan (MWAS, sometimes referred to as EWAS which stands for Epigenome-Wide Association Scan) to identify DNA methylation changes in relation to the presence/absence of the *F5* rs6025 mutation in 349 (98/251) MARTHAVT patients. Main findings were replicated in an independent study of 214 (53/161) individuals, processed with the same Illumina array.

## Material and Methods

### Ethics Statement

For MARTHA, ethics approval was obtained from the "Département santé de la direction générale de la recherche et de l'innovation du ministère" (Projects DC: 2008-880 & 09.576).

For the F5L-families study, ethics approval was obtained from the Ottawa Hospital and the University of Toronto ethics boards. All subjects in both studies provided written informed consent in accordance with the Declaration of Helsinki.

### Study populations

#### Discovery study sample

The MARTHA study is a collection of 1,542 patients with VT recruited from the Thrombophilia centre of La Timone hospital (Marseille, France) [27]–[30]. All subjects had a documented history of VT, were free of chronic conditions, and were free of inherited thrombophilia including: anti-thrombin, protein C and protein S deficiencies and homozygosity for the Factor V Leiden and Factor II G20210A mutations. For the current project, 349 MARTHA patients were randomly selected for DNA methylation analysis.

#### Replication study sample

The family study is composed of five extended French-Canadian pedigrees, totaling 255 relatives, ascertained at the Thrombosis Clinic of the Ottawa Hospital through single probands with idiopathic VT and heterozygote for the Factor V Leiden mutation. Probands were free of acquired VT risk factors such as cancer, myeloproliferative disease, pregnancy, puerperium, prolonged immobilization, trauma, surgery and antiphospholipid syndrome, and were free of inherited thrombophilia (see above). A detailed description of this study can be found in [27]. Only 218 family members for whom DNA was still available were included in the current work.

### Genome wide DNA methylation assay

Genomic DNA was isolated from peripheral blood cells using an adaptation of the method proposed by [31]. For each sample, 1 µg genomic DNA was bisulphite converted using the Qiagen EpiTect 96 Bisulfite Kit. Then, 200 ng of bisulfite-converted DNA at 50 ng/µl was independently amplified, labeled, and hybridized to Infinium HumanMethylation450 BeadChip microarrays [25] and scanned with default settings using the Illumina iScan. This Illumina array covers 99% of RefSeq genes and surveys the DNA methylation levels at 482,421 CpG sites, with an average of 17 CpG sites per gene region. The discovery and replication samples were processed simultaneously at The Center for Applied Genomics (TCAG, Toronto, Canada).

### Quality controls and normalization procedures

From the 485,577 probes available on the Illumina array, we excluded from further analyses probes that measured single nucleotide polymorphisms (n = 65), that are either cross-reactive (n = 30,969) or polymorphic at the targeted CpG site (n = 66,877) [32], [33]. Of note, 4,464 probes shared the two last features.

DNA methylation data were expressed as a β-value, a continuous variable over the [0–1] interval, representing the percentage of methylation of a given CpG site [34]. Methylation values were corrected for background by use of the Noob method implemented in the "methylumi" package [35], for dye bias following the manufacturer's recommendation (http://support.illumina.com/downloads/genomestudio_m_module_v18_ug_%2811319130_b%29.ilmn) and normalized for design type bias according to the SWAN method [36] implemented in the minfi R package [37].

Quality control and normalization were done simultaneously on the MARTHA and F5L-families datasets. Probes (n = 4,010) with a detection p-value (as described in the "minfi" package) greater than 0.05 in more than 5% of the total processed samples were then excluded from further analyses. Principal components analysis was carried out on probe data to detect outliers and four F5L-families individuals were then excluded. This led to a final selection of 388,120 probes (among which 1,289 tagged for CpH sites) that were tested for association with the presence/absence of the *FV* Leiden mutation-tagging rs6025-C allele.

### Biological measurements

In MARTHA, we used the Agkistrodon contortrix venom (ACV) test as a quantitative biomarker of the protein C pathway. The ACV test was expressed as a normalized ACV value (ACVn) as described in [30]. The ACVn ratio was available in 260 MARTHA patients with DNA methylation measurements. A complete blood count, including white blood cell types (neutrophils, lymphocytes, monocytes, eosinophils, and basophils), was determined by ADVIA 120 Hematology System (Siemens Healthcare Diagnostics, Deerfield, IL).

In F5L-families, activated protein C resistance (APCR) levels were determined in 208 individuals using the APC-aPTT assay. The results of the test are expressed as the APC-sensitivity ratio, which is the quotient of the activated partial thromboplastin time (aPTT) of the plasma sample with and without exogenous APC [38].

### Genotyping

MARTHA patients were genotyped with the Illumina Human 610/660W-Quad beadchips [28]. Autosomal SNPs that satisfied quality control criteria (n = 481,002) [39] were then used for imputing SNPs from the 1000 Genomes 2012-02-14 release reference dataset. Imputation was performed by use of MACH (v1.0.18.c) software [40]. All SNPs with acceptable imputation quality (r^2^>0.3) [41], minor allele frequency>0.01 and mapping to the chromosome 1 169101258–169555769 locus were tested for association with *SLC19A2* probes.

The F5L-families study was genotyped with the Illumina 660W-Quad beadchip. Detailed description of the quality control procedure is available in [28].

### Statistical Analysis

#### Discovery MWAS

Because methylation β-values are often not normally distributed, exhibiting bi-modality, right-, or left-tailed skewed distributions, our discovery MWAS was performed using a logistic regression model with carrier status (yes or no) as the outcome and the β-values as covariates. Any methylation probes that satisfied the Bonferroni threshold of 1.29×10^−7^ (∼0.05/388,120) were selected and their distribution was assessed (Figure S1). For uni-modal probes, a linear regression model was also applied with β-values as the outcome and carrier status as the covariate to assess the consistency of the MWAS results and to provide an estimate of the effect of the rs6025 variant on the DNA methylation β-value. A linear regression model with M-transformed values [42], [43] instead of β-values as outcome was also applied to provide further statistical support to the obtained results (Table S1). Analyses were adjusted for age, sex, batch and chip effects [44]. Because DNA methylation levels measured in peripheral blood DNA reflect the average level of DNA methylation in different cell types including lymphocytes, neutrophils, basophils and eosinophils, all analyses were also adjusted for cell type composition to avoid any contamination bias [45]–[47]. For this, we used specific biological counts of lymphocytes, monocytes, neutrophils, eosinophils and basophils available for all MARTHA samples to characterize cell type composition.

#### Replication study

In the F5L-families study, association of selected probes with rs6025 was assessed using the linear model mentioned above, after having checked for the uni-modality of the data distribution (Figure S1). In order to handle correlations between family data, a linear mixed regression model as implemented in the NMLE R package (http://cran.r-project.org/web/packages/nlme/) was employed where the family variable was defined as a random effect. Analyses were adjusted for age, sex, batch, chip and cell type composition. As specific cell type counts were not available in the family study, adjustment for cell type composition was handled by the method described in [48], [49]. The Bonferroni corrected threshold of 0.0167 ( = 0.05/3) was used for declaring replication.

#### Further analyses

Association of selected probes with quantitative biomarkers was tested using a linear (mixed in F5L-families) model where log-transformed biomarker values were used as the outcome and the methylation β-values as covariates. Models were adjusted by the same covariates as described above.

The association of imputed SNPs with methylation β-values was tested by entering the allele dosage of the imputed SNP as a covariate in a linear regression model with β-values as the outcome. The allele dosage is a real number ranging from 0 to 2 corresponding to the expected number of minor alleles computed from the posterior probabilities of possible imputed genotypes.

To get more power for detecting CpG sites variability associated with the *F5* rs6025, we finally performed a combined analysis of both MARTHA and F5L-families studies. For this part, linear regression analyses (mixed linear model in F5L-families) were conducted for each CpG β-value with the rs6025 as covariate while adjusting for the same variables as indicated above. Regression coefficients associated with the rs6025 were then combined into a random-effect meta-analysis using the GWAMA program [50].

## Results

Brief characteristics of the two studied populations are given in Table 1. To support the validity of the discovery MARTHA DNA methylation dataset, we investigated two previously reported robust associations with DNA methylation marks, the association of smoking with decreased methylation levels at *F2RL3* CpG cg03636183 [16], [22] and the association of rs713586 with DNAJC27/ADCY3 CpG cg01884057 [51]. Consistent and strong significant associations were observed in MARTHA. Current smokers exhibited lower levels of methylation at cg03636183 compared to non-smokers and former smokers (p = 1.13×10^−29^) (Figure S2). The rs713586-T allele was associated with decreased methylation β-values at cg01884057 in a fairly additive manner (p = 7.38×10^−68^) (Figure S3).

**Table 1:** Characteristics of the studied populations.

	MARTHA	F5L-famiiles
	N = 349	N = 214
Mean age in yrs ± SD	43.8±14.1	39.6±16.7
Males/Females	75/274	101/113
VT patients (%)	349 (100%)	11 (5.1%)
Heterozygote carriers of the *F5* rs6025	98	53
ACVn ratio(1)	0.89±0.38	NA
*F5* rs6025 carriers	0.52±0.10	NA
Non-carriers	1.09±0.32	NA
APCR ratio (2)	NA	2.56±0.67
*F5* rs6025 carriers	NA	1.67±0.19
Non-carriers	NA	2.86±0.49

(1) In MARTHA, ACVn ratio was significantly (p = 1.63 10^−38^) decreased in *F5* rs6025 carriers compared to non-carriers.

(2) In families, APCR ratio was significantly (p = 9.98 10^−47^) decreased in *F5* rs6205 carriers compared to non-carriers.

**Figure 1:**
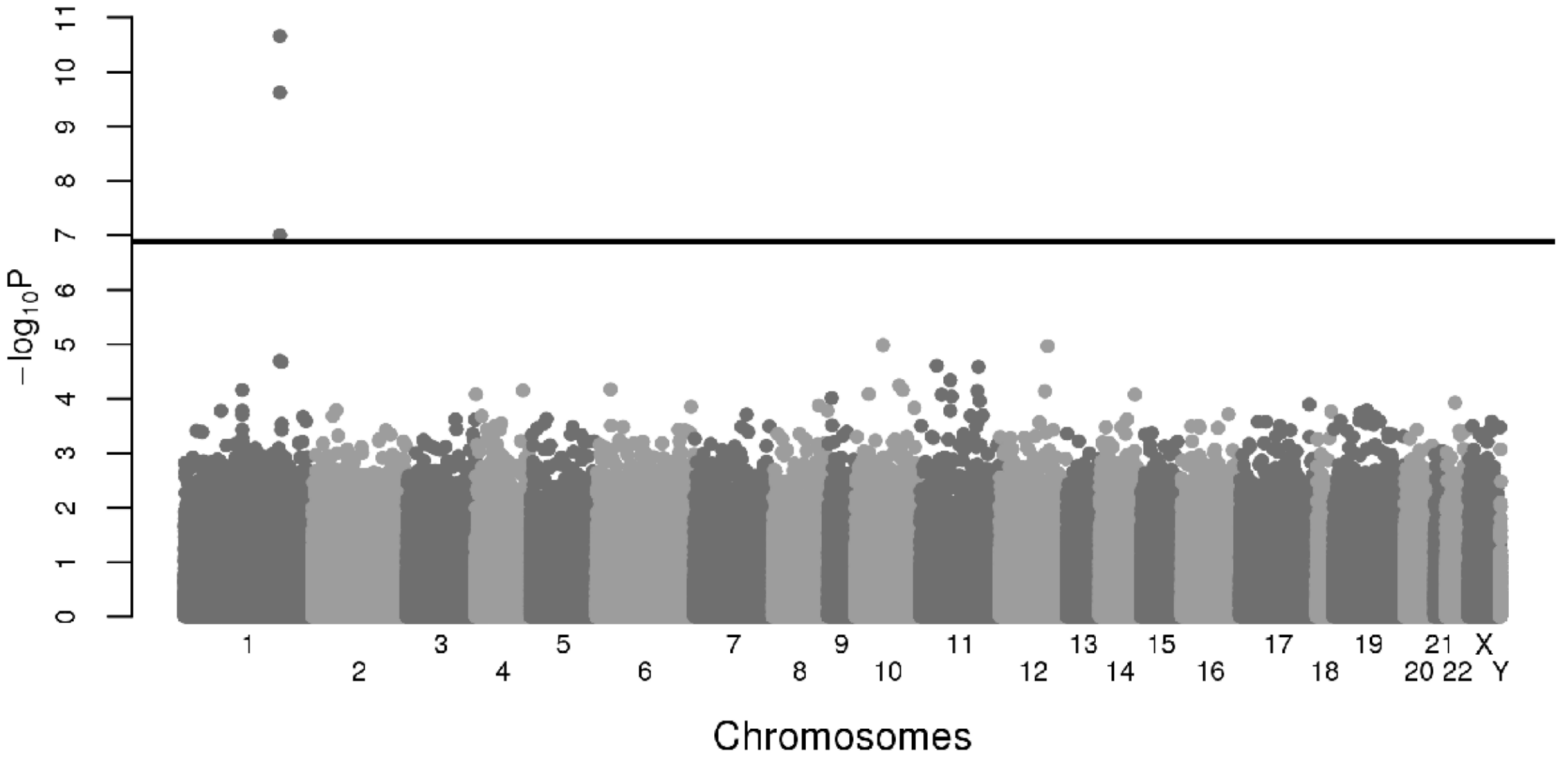
Manhattan plot of the MWAS results at 388,120 CpG sites.

A Manhattan plot of the MWAS results is shown in Figure 1. Three CpG sites, all mapping the *SLC19A2* gene region, were associated with rs6025 at the genome-wide significant threshold of 1.29×10^−7^ (∼0.05/388,120). DNA methylation levels at these sites, cg16548605 (p = 3.61×10^−11^), cg04083076 (p = 2.82×10^−10^) and cg09671955 (p = 2.66×10^−8^), were decreased in carriers of the rs6025-C allele compared to non-carriers (Table 2, Figure S4). Of note, the strongest association observed with any *F5* CpG site was p = 0.045 for cg16054275.

**Table 2:** Association(1) of *SLC19A2*CpG sites with rs6025 (FV Leiden mutation) in the discovery and replication studies.

	Discovery MARTHA study			Replication F5L-families study		
	Non-Carriers (N = 251)	Carriers (N = 98)	Association Test p-value(2)	Non-Carriers (N = 161)	Carriers (N = 53)	Association Test p-value(2)
cg16548605	0.93 (0.02)	0.89 (0.03)	1.90 10^−29^	0.93 (0.02)	0.90 (0.03)	6.58 10^−14^
cg04083076	0.73 (0.06)	0.64 (0.06)	5.73 10^−22^	0.74 (0.06)	0.67 (0.09)	1.19 10^−10^
cg09671955	0.53 (0.06)	0.48 (0.07)	3.49 10^−12^	0.55 (0.07)	0.50 (0.07)	5.62 10^−7^

(1) Association is expressed as methylation β-value mean (SE) in carriers and non-carriers.

(2) Reported p-values were those derived from a linear regression model where the probe methylation level was the outcome and the carrier status the covariate, while adjusting for age, sex, batch, chip and cell type composition.

These three CpG probes were tested for replication in 214 related individuals from the F5L Thrombophilia French-Canadian Pedigree study [27], [28], referred thereafter to as the F5L-families study. In this independent study, all three *SLC19A2* probes also exhibited lower DNA methylation levels in carriers (n = 53) compared to in non-carriers (n = 161) of the rs6025-C allele (Table 2; Figure S5).

To further validate these results, using a linear model, we assessed the association of the 3 *SLC19A2* probes with quantitative biomarkers of the Protein C pathway known to be under the strong influence of rs6025: the Agkistrodon contortrix venom test (ACVn) [30], [52] in the discovery MARTHA population and the activated protein C resistance (APCR) test [53] in the replication family study. In both studies, these biomarkers demonstrated decreased levels in carriers of the *F5* rs6025-C allele compared to non-carriers (Table 1). The three *SLC19A2* CpG sites were significantly associated with the two biomarkers, with all p-values<10^−3^ (Table 3). For example, every 0.1 unit increase in the methylation β-value at cg16548605 was associated with a 46.9% (95% confidence interval: 32.6%–61.1%) higher ACVn value in the MARTHA population and with a 50.0% (95%CI: 36.3%–63.7%) higher APCR value in the F5L-families. After adjusting for rs6025, these associations completely vanished, with all p-values>0.01 (Table 3).

**Table 3:** Association(1) of *SLC19A2* CpG sites with ACVn (MARTHA) and APCR (F5L-families) phenotypes.

	MARTHA study (N = 260)		F5L-families study (N = 208)	
	raw(2)	Adjusted for rs6025	raw(2)	Adjusted for rs6025
cg16548605	46.9 (32.6–61.1) p = 8.14 10^−10^	−1.2 (−14–11.7) p = 0.86	58.1 (44–72.1) p = 1.11 10^−13^	13.6 (3.1–24.1) p = 0.01
cg04083076	18.1 (11.5–24.7) p = 2.12 10^−7^	−3.1 (−8.8–2.50) p = 0.28	21.9 (15.1–28.7) p = 2.13 10^−9^	3.9 (−0.8–8.5) p = 0.10
cg09671955	15.1 (7.4–22.9) p = 1.7 10^−4^	−1.9 (−7.8–4.0) p = 0.53	14.4 (7.3–21.4) p = 1.02 10^−4^	−1.3 (−4.6–4.1) p = 0.90

(1) Association is expressed as % change in phenotype (95% Confidence Interval) for every 0.1 unit increase in methylation β-value.

(2) Analysis were adjusted for age, sex, batch, chip and cell type composition.

**Figure 2:**
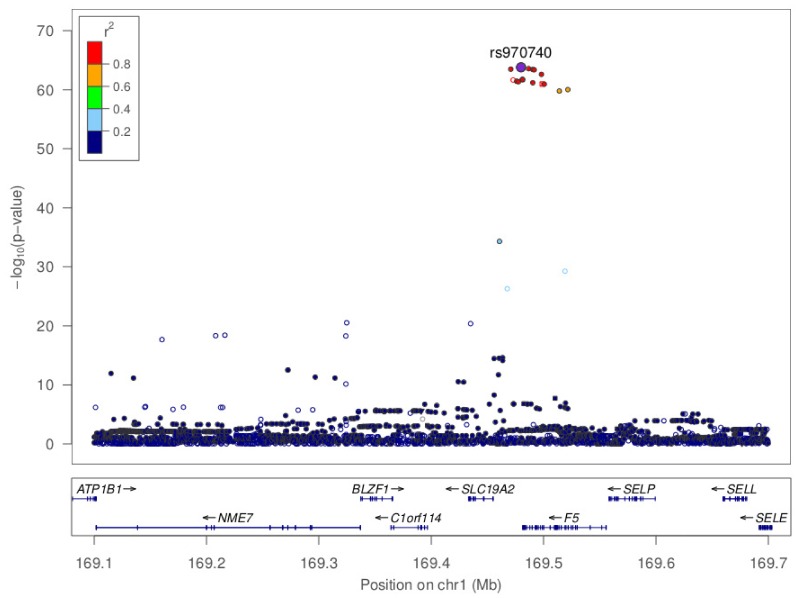
Region Association plot of the association between chromosome 1q23.3 SNPs and cg16548605 CpG site variability in the MARTHA study.

Because the *SLC19A2* gene maps to chromosome 1q23.3 in the vicinity of the *F5* gene, a locus known to exhibit strong linkage disequilibrium (LD) over a large genomic distance of ∼460 Kb [39] (Figure S6), one cannot rule out the possibility that the associations between rs6025 and methylation at *SLC19A2* probes were due to other SNPs in LD with rs6025. We therefore examined the association of the methylation levels of the three *SLC19A2* probes with 3,213 SNPs at this locus using genome-wide SNP data available in the MARTHA study. Results of these association analyses, where DNA methylation levels were the outcome and the SNPs the predictors, are illustrated in Figure 2. The strongest association for cg16548605 was observed with rs970740 (p = 1.61×10^−66^) where the minor C allele was associated with decreased cg16548605 methylation levels (Table 4) (regression coefficient for adjusted allele effect β = −0.049±0.0022). The same pattern of associations was observed in the F5L-families study (Table 4). The C allele was also associated with decreased ACVn values (β = -0.415±0.043, p = 3.20×10^−18^) (Table 5). Interestingly, in a joint model where both rs970740 and rs6025 were used as covariates for predicting cg16548605 variability, the effect of rs970740 was highly significant (p = 1.05×10^−38^) but that of rs6025 was not (p = 0.90). Conversely, in a similar joint model for predicting ACVn levels, only the effect of rs6025 was significant (p = 1.65×10^−20^) while the effect of rs970740 completely vanished (p = 0.79). Rs970740 lies in the upstream *SLC19A2*/downstream *F5* region and is in moderate LD (r^2^ = 0.65) with the *F5* rs6025. Similar patterns were observed for the other *SLC19A2* cg04083076 and cg09671955 CpG sites (data not shown).

**Table 4:** Association of rs970740 with *SLC19A2* cg1658605, cg0483076 and cg09671955 levels.

	MARTHA				F5L-families study			
	TT (N = 232)	TC (N = 114)	CC (N = 3)	P value	TT (N = 140)	TC (N = 68)	CC (N = 3)	P value
cg16548605	0.935 (0.010)	0.889 (0.027)	0.797 (0.045)	1.66×10^−66^	0.930 (0.010)	0.900 (0.029)	0.845 (0.034)	4.49×10^−33^
cg04083076	0.735 (0.051)	0.642 (0.056)	0.541 (0.135)	1.16×10^−34^	0.752 (0.055)	0.681 (0.077)	0.607 (0.142)	1.65×10^−20^
cg09671955	0.538 (0.059)	0.479 (0.066)	0.403 (0.063)	8.00×10^−17^	0.556 (0.066)	0.503 (0.071)	0.490 (0.053)	2.76×10^−10^

p-values were adjusted for age, sex, batch, chip and cell type composition.

In the F5L-families study, the rs970740 was not genotyped but substituted by the proxy rs2420371 that is in complete association (r^2^ = 1) with it.

**Table 5:** Association of rs970740 and rs6025 with *SLC19A2* cg16548605 CpG and ACVn levels in the MARTHA study.

	cg16548605	ACVn (log)
Univariate analysis ^(1)^		
rs970740	β = −0.049 (0.0022) p = 1.61 10^−66^	β = −0.415 (0.043) p = 3.20 10^−18^
rs6025	β = −0.044 (0.0035) p = 1.9 10^−29^	β = −0.653 (0.042) p = 3.58 10^−37^
Joint analysis ^(2)^		
rs970740	β = −0.050 (0.0033) p = 1.05 10^−38^	β = −0.014 (0.052) p = 0.79
rs6025	β = 0.001 (0.004) p = 0.90	β = −0.641 (0.062) p = 1.65 10^−20^

Association is expressed as the additive effect of the minor alleles on the variability of cg16548605 and log ACVn (95% Confidence Interval) adjusted for age, sex, batch, chip and cell type composition. In the univariate analysis^(1)^, one SNP at a time is used as a covariate for predicting the phenotype. In the joint analysis ^(2)^, both SNPs are simultaneously introduced as predictors in the linear regression models.

These results demonstrate that two independent phenomena act at this locus: an effect of rs970740 (or its proxies) on the variability of *SLC19A2* methylation levels and the effect of *F5* rs6025 on the ACVn biomarker. The presence of LD between rs970740 and the *F5* rs6025 mutation confounds the associations between methylation at *SLC19A2* sites and both the *F5* rs6025 and the ACVn biomarker.

To improve statistical power and increase opportunity to detect smaller effect sizes of additional CpG sites and *F5* rs6025 associations, we combined the discovery and replication study samples into a meta-analysis. An additional CpG probe (cg26009832) mapping the *SLC19A2/F5* locus reached genome-wide significance (p = 1.42 10^−8^).

## Discussion

The starting hypothesis of this work was that DNA methylation marks associate with the *F5* rs6025 mutation and contribute to the incomplete penetrance of this strong genetic risk factor for VT. Thus, we undertook the first MWAS of the *F5* rs6025 in a large sample of 349 individuals and replicated the findings in an independent sample of 214 related subjects. We identified and replicated three CpG sites exhibiting a genome-wide significant difference in methylation levels in carriers and non-carriers of the mutation. These CpG sites were also strongly associated with the plasma variability of quantitative biomarkers influenced by the *F5* rs6025. However, when we integrated our MWAS and GWAS data, the observed associations between methylation levels at three CpG sites in *SLC19A2* and *F5* rs6025 were in fact due to LD between the rs6025 and SNPs located in *SLC19A2*.

We observed strong statistical evidence for association between the *SLC19A2* promoter rs970740 SNP (or any SNP in strong LD with it) and three identified *SLC19A2* CpG sites, independently of *F5* rs6025. According to public database, including 1000 Genomes, none of the probes measuring these three CpG sites are polymorphic and the rs970740 does not map to a CpG island. This strongly suggests the existence of variant(s) influencing the variability of DNA methylation levels at the *SLC19A2* gene. How the rs970740 T/C genetic variation (or any linked SNP) affects *SLC19A2* DNA methylation remains an open question. This could be through the creation of a transcription factor binding site, the modification of the local CpG sites distribution, or more complex phenomena [54]–[59]. The *SLC19A2* gene codes for a thiamine transporter protein that has been associated with human anemia syndrome [60]–[62]. Our results suggest that genetically determined DNA methylation sensitive mechanisms are involved in this disease susceptibility.

Several conclusions could be drawn from this work. First, three identified CpG sites were found to be strongly associated with the plasma variability of two quantitative biomarkers of the coagulation cascade, supporting the potential of genome-wide DNA methylation data to identify epigenetic marks associated with biological phenotypes involved in thrombotic disorders. Nonetheless, this works highlights the need for careful analyses of associations between genetic variants, biological phenotypes, and methylation at CpG sites to avoid false inference on functional variant(s), in particular due to LD extending over large genomic distances. Integrating MWAS, GWAS and biological data from the same individuals, as illustrated here, is key to elucidating these relationships. Second, if such cautions are taken, DNA methylation data can help to dissect the functional mechanisms associated with known disease-causing SNPs.

Several limitations must be acknowledged. First, the design of our study may not be optimal. As we did not have access to a case-control study for VT with genome-wide DNA methylation data, we adopted a 'case-only' approach for our discovery stage. Such approach has been shown to be a valid alternative to detect gene × environment or gene × gene interactions [63], [64]. We here used this strategy with the aim of identifying epigenetic factors that interact with the FV Leiden mutation to modulate the risk of VT. Since, in our replication study, 45 carriers were VT patients and the remaining 8 carriers were healthy individuals, we also looked into this dataset for specific methylation patterns associated with VT but the low sample size precludes from identifying any significant association (data not shown).

Second, because homozygosity for *FV* Leiden mutation was an exclusion criteria for the MARTHA study and no homozygote was observed in the F5L-families, our analysis only included heterozygous carriers which may have reduced our power to identify CpG sites under the strong influence of the mutation.

Third, while extremely dense, the used Illumina array does not cover all sites of the genome that could be subject to DNA methylation, we cannot exclude that some relevant methylation association has been missed.

Fourth, the sample size of our discovery study was large enough to detect, at the genome-wide level of 1.29 10^−7^, a 0.05 increase in the methylation β-value. Whether an increase of smaller magnitude in DNA methylation marks detected in whole blood could be biologically relevant remains an open question. Whole blood DNA methylation levels reflect the average levels resulting from the epigenetic state at different white blood cells. Therefore, the cell subtype and tissue specific methylation marks would show a weaker effect in whole blood compared to levels that could be measured in thrombosis-relevant effector cells (e.g. monocytes, endothelial cells, hepatocytes). This phenomenon was recently observed and discussed for other cardiovascular-related phenotypes [12], [65]. Thus, we cannot rule out the possibility that a stronger influence of *F5* rs6025on DNA methylation levels exists in specific cell types or tissues, such as the liver where F5 is mainly synthetized.

Last, we observed evidence that *SLC19A2*, with genetically determined DNA methylation levels, is a methylation quantitative trait locus (mQTL). However, as we did not have access to gene expression data, we were not able to assess whether the observed genetic influence on *SLC19A2* DNA methylation levels is followed by a direct impact on *SLC19A2* expression. Further epi-mapping at this locus would be of great interest.

In conclusion, our work does not support the existence of DNA methylation marks that could explain the incomplete penetrance of the *F5* rs6025. The incomplete penetrance could be the result of complex haplotype effects at the *F5* locus, or interaction at this locus with other genetic or environmental exposures; such investigations would require alternative study designs and much larger sample sizes to detect effects.

This work does, however, illustrate the promises and pitfalls of MWAS on peripheral blood DNA in large epidemiological studies, and suggests that the anemia-associated *SLC19A2* gene is a mQTL.

## Supporting Information

Figure S1 **Density distributions of** ***SLC19A2*** **methylation probes in the MARTHA and F5L-families studies.**
(PDF)

Figure S2 **Association of smoking with methylation β-values at** ***F2RL3*** **CpG cg03636183 in the MARTHA study.**
(PDF)

Figure S3 **Association of rs713586 with methylation β-values at CpG site cg01884057 in the MARTHA study.**
(PDF)

Figure S4 **Boxplot of the association between the** ***F5*** **rs6025-C allele with** ***SLC19A2*** **methylation probes in the discovery MARTHA study.**
(PDF)

Figure S5 **Boxplot of the association between the** ***F5*** **rs6025-C allele with** ***SLC19A2*** **methylation probes in the replication F5L-families study.**
(PDF)

Figure S6 **Linkage Disequilibrium plot at the 1q23.3 locus in the MARTHA study.** This plot was drawn with the Haploview software (*Barrett JC, Fry B, Maller J, Daly MJ. Haploview: analysis and visualization of LD and haplotype maps. Bioinformatics. 2005 [PubMed ID: 15297300]*).
(PDF)

Table S1 **Consistency between the statistical p-values derived from the linear analyses of β and M-transformed values.**
(DOCX)
