# Supplementary material for: Genome-Wide Investigation of DNA Methylation Marks Associated with FV Leiden Mutation
Source: PLoS One. 2014 Sep 29;9(9):e108087. doi: 10.1371/journal.pone.0108087 (PMC4179266; doi:10.1371/journal.pone.0108087)
Supplement: Figure S1 — Density distributions of SLC19A2 methylation probes in the MARTHA and F5L-families studies. (PDF) [file pone.0108087.s001.pdf]

Supplementary Figure 1 - Density distributions of the identified *SLC19A2* methylation probes in the MARTHA (N = 349) and F5L-families studies (N = 214)

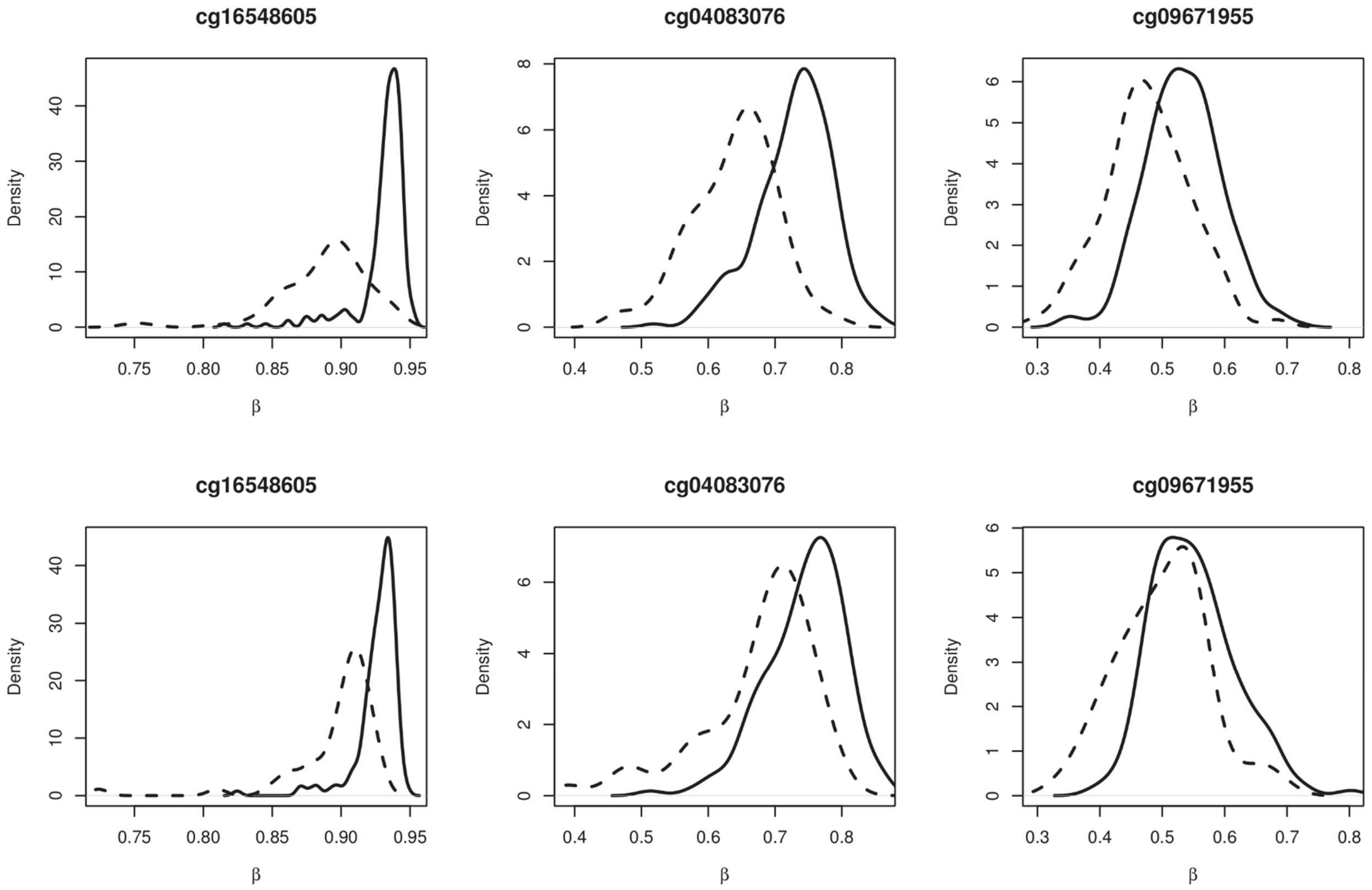

Shown distributions correspond to that of the *SLC19A2* cg16548605 (left), cg04083076 (middle) and cg09671955 (right) observed in MARTHA (top) and F5L-families (bottom) in carriers (dashed line) and non-carriers (straight line) of the *F5* rs6025-C allele.
