# Supplementary figures and images for: Genome-Wide Investigation of DNA Methylation Marks Associated with FV Leiden Mutation
Source: PLoS One. 2014 Sep 29;9(9):e108087. doi: 10.1371/journal.pone.0108087 (PMC4179266; doi:10.1371/journal.pone.0108087)

**Supplementary Figure 2** - Association of smoking with *F2RL3* CpG cg03636183 in the MARTHA study

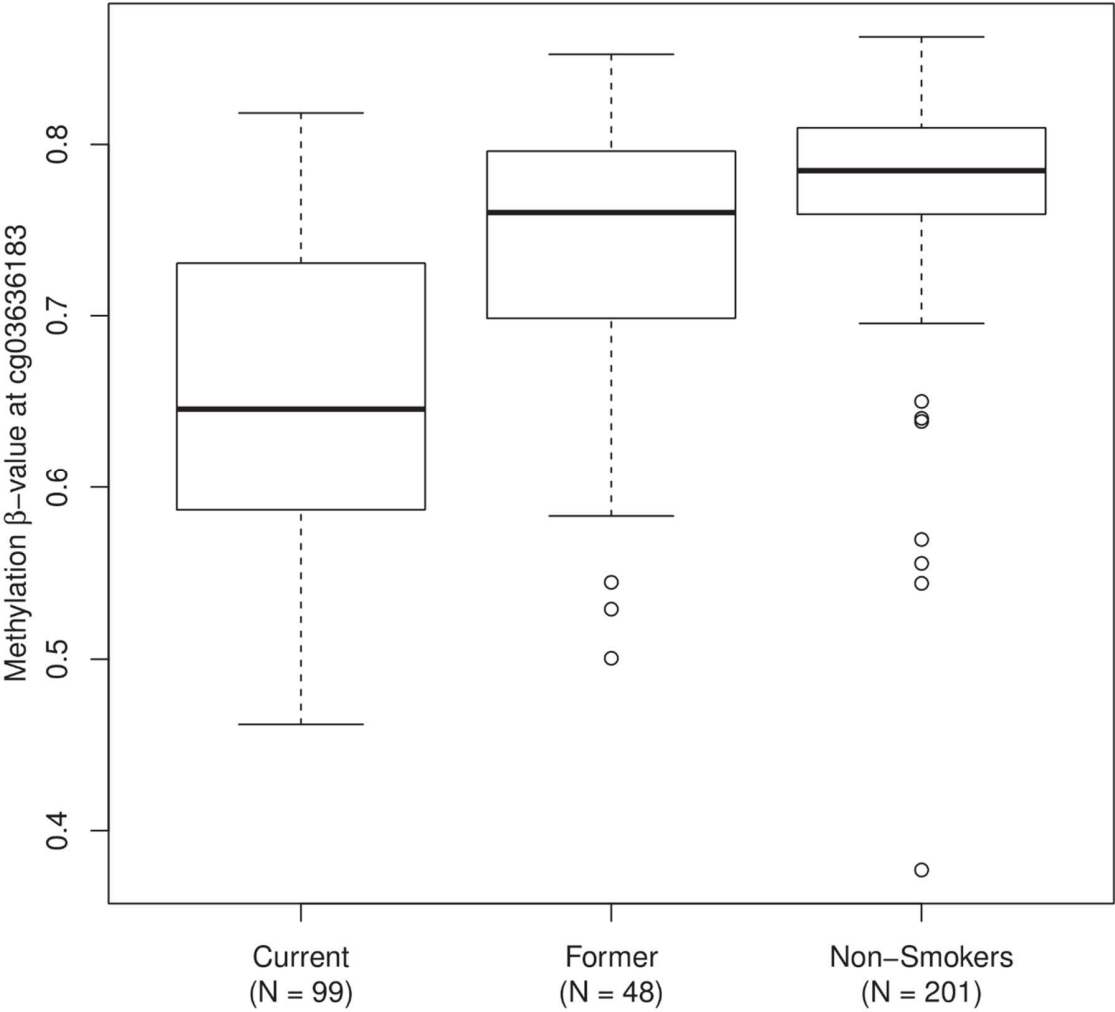

Supplement: Figure S2 — Association of smoking with methylation β-values at F2RL3 CpG cg03636183 in the MARTHA study. (PDF) [file pone.0108087.s002.pdf]

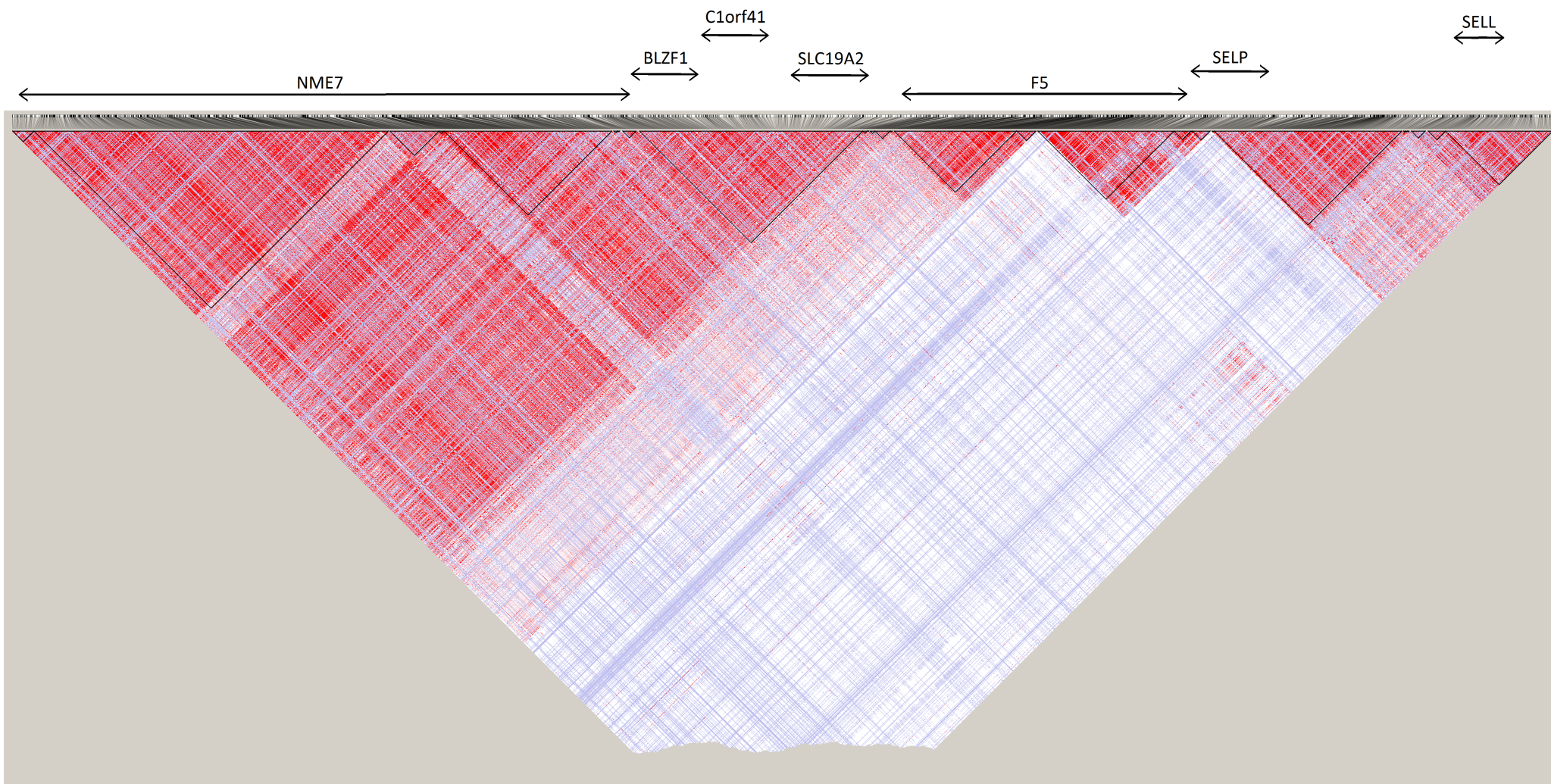

Supplement: Figure S6 — Linkage Disequilibrium plot at the 1q23.3 locus in the MARTHA study. This plot was drawn with the Haploview software (Barrett JC, Fry B, Maller J, Daly MJ. Haploview: analysis and visualization of LD and haplotype maps. Bioinformatics. 2005 [PubMed ID: 15297300]). (PDF) [file pone.0108087.s006.pdf]
