# Supplementary material for: Genome-Wide Investigation of DNA Methylation Marks Associated with FV Leiden Mutation
Source: PLoS One. 2014 Sep 29;9(9):e108087. doi: 10.1371/journal.pone.0108087 (PMC4179266; doi:10.1371/journal.pone.0108087)

Supplementary Figure 3 - Association of rs713586 with methylation β-values at CpG site cg01884057 in the MARTHA study

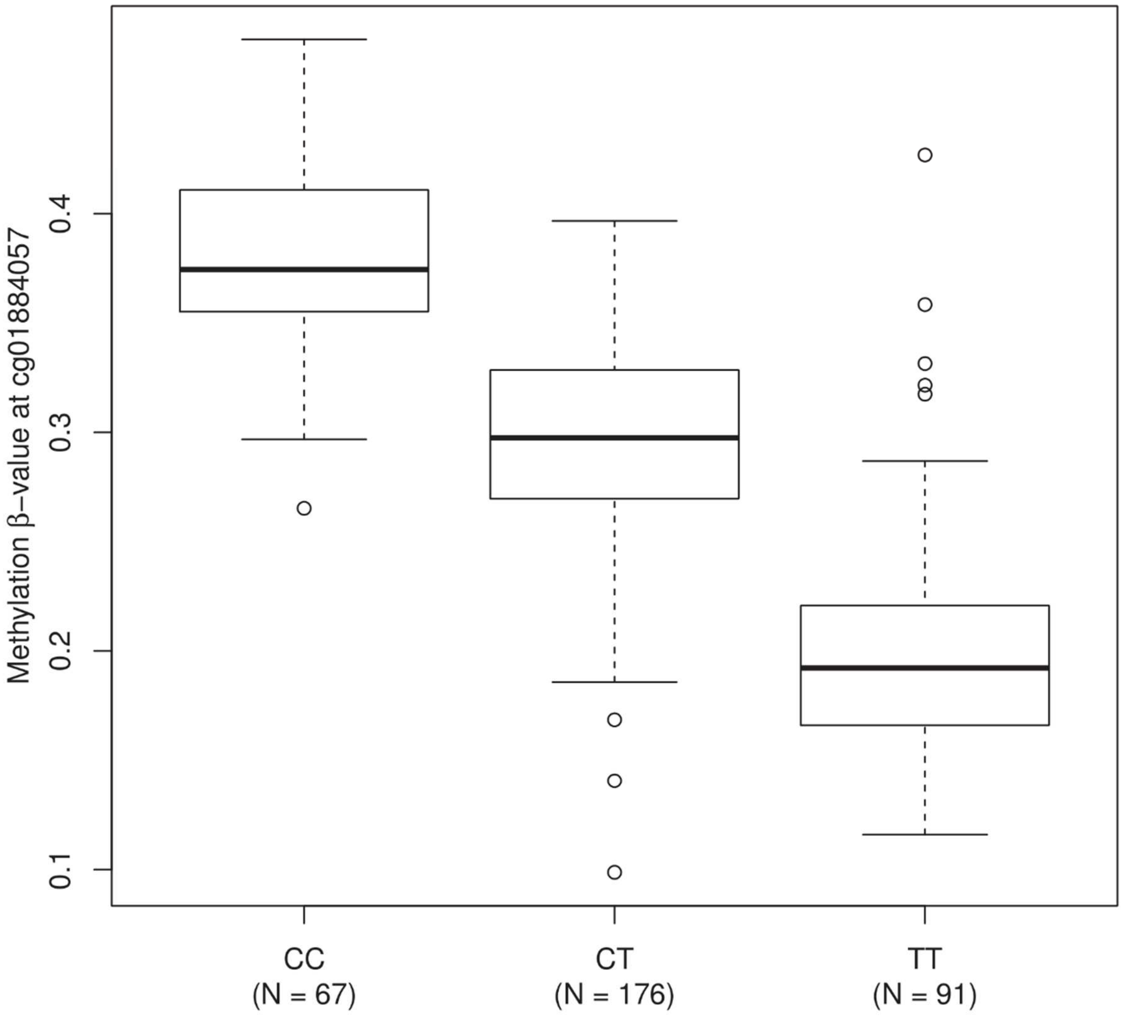

Supplement: Figure S3 — Association of rs713586 with methylation β-values at CpG site cg01884057 in the MARTHA study. (PDF) [file pone.0108087.s003.pdf]
