# Supplementary material for: Genome-Wide Investigation of DNA Methylation Marks Associated with FV Leiden Mutation
Source: PLoS One. 2014 Sep 29;9(9):e108087. doi: 10.1371/journal.pone.0108087 (PMC4179266; doi:10.1371/journal.pone.0108087)

**Supplementary Figure 4** - Boxplot representation of the association of *F5* rs6025-C allele with the identified *SLC19A2* methylation probes in the discovery MARTHA cohort

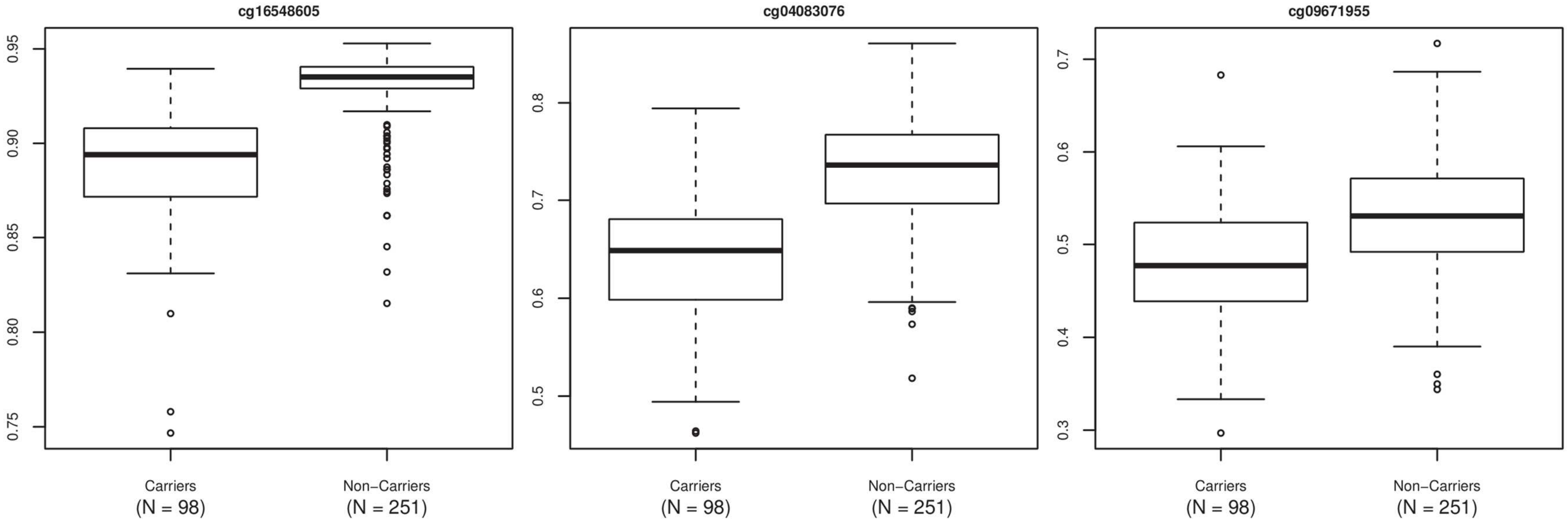

Supplement: Figure S4 — Boxplot of the association between the F5 rs6025-C allele with SLC19A2 methylation probes in the discovery MARTHA study. (PDF) [file pone.0108087.s004.pdf]
