# Supplementary material for: Genome-Wide Investigation of DNA Methylation Marks Associated with FV Leiden Mutation
Source: PLoS One. 2014 Sep 29;9(9):e108087. doi: 10.1371/journal.pone.0108087 (PMC4179266; doi:10.1371/journal.pone.0108087)
Supplement: Table S1 — Consistency between the statistical p-values derived from the linear analyses of β and M-transformed values. (DOCX) [file pone.0108087.s007.docx]

Supplementary Table S1- Consistency between the statistical p-values derived from the linear analyses of β and M-transformed values

| Results from Table2 | Analysis on β values | | Analysis on M-transformed values | |
| --- | --- | --- | --- | --- |
|  | MARTHA | F5L | MARTHA | F5L |
| cg16548605 | 1.90 10^-29^ | 6.58 10^-14^ | 3.90 10^-34^ | 1.61 10^-18^ |
| cg04083076 | 5.73 10^-22^ | 1.19 10^-10^ | 4.82 10^-21^ | 1.08 10^-10^ |
| cg09671955 | 3.49 10^-12^ | 5.62 10^-7^ | 4.21 10^-12^ | 9.76 10^-7^ |

| Results from Table4 | Analysis on β values | | Analysis on M-transformed values | |
| --- | --- | --- | --- | --- |
|  | MARTHA | F5L | MARTHA | F5L |
| cg16548605 | 1.66 10^-66^ | 4.49 10^-33^ | 6.50 10^-80^ | 7.53 10^-43^ |
| cg04083076 | 1.16 10^-34^ | 1.65 10^-20^ | 3.13 10^-33^ | 1.31 10^-20^ |
| cg09671955 | 8.00 10^-17^ | 2.76 10^-10^ | 9.91 10^-17^ | 6.75 10^-10^ |

| Results from Table5 | Analysis on β values | | Analysis on M-transformed values | |
| --- | --- | --- | --- | --- |
|  | Univariate analysis | Joint analysis | Univariate analysis | Joint analysis |
|  |  |  |  |  |
| rs970740 | 1.61 10^-66^ | 1.05 10^-38^ | 6.50 10^-80^ | 2.03 10^-47^ |
| rs6025 | 1.90 10^-29^ | 0.90 | 3.90 10^-34^ | 0.90 |
